# The Evolution of Food Calls: Vocal Behaviour of Sooty Mangabeys in the Presence of Food

**DOI:** 10.3389/fpsyg.2022.897318

**Published:** 2022-06-30

**Authors:** Fredy Quintero, Sonia Touitou, Martina Magris, Klaus Zuberbühler

**Affiliations:** ^1^Department of Comparative Cognition, Institute of Biology, Université de Neuchâtel, Neuchâtel, Switzerland; ^2^Centre Suisse de Recherches Scientifiques, Taï Monkey Project, Abidjan, Côte d’Ivoire; ^3^School of Psychology and Neurosciences, University of St Andrews, St Andrews, United Kingdom

**Keywords:** food-associated calls, *Cercocebus atys*, vocal communication, grunt, close-range vocalisations

## Abstract

The two main theories of food-associated calls in animals propose functions either in cooperative recruitment or competitive spacing. However, not all social animals produce food calls and it is largely unclear under what circumstances this call type evolves. Sooty mangabeys (*Cercocebus atys*) do not have food calls, but they frequently produce grunts during foraging, their most common vocalisation. We found that grunt rates were significantly higher when subjects were foraging in the group’s periphery and with small audiences, in line with the cooperative recruitment hypothesis. In a subsequent field experiment we presented highly desired food items and found that discovering individuals called, unless harassed by competitors, but that the calls never attracted others, confirming that the grunts do not convey any information referential to food. Our data thus suggest that the evolution of cooperative food calling is a two-step process, starting with increased motivation to vocalise in the feeding context, followed by the evolution of acoustic variants derived from context-general contact calls. This evolutionary transition may only occur in species that feed on clumped, high-quality resources where social feeding is competitive, a condition not met in sooty mangabeys.

## Introduction

Food-associated calls have been reported in many socially living avian and mammalian species (Clay et al., 2012), produced when individuals find food or during feeding (Elowson et al., 1991; Valone, 1996; Slocombe and Zuberbühler, 2006; Clay et al., 2012). The behaviour is interesting because it raises basic questions about signal evolution and call meaning, as well as the evolution of cooperative behaviour more generally. For example, one theoretically relevant line of enquiry has focussed on whether food calls qualify as ‘functionally referential’ signals, that is, whether they inform others about a receiver-relevant event experienced by the caller (e.g., Hauser and Marler, 1993a,b; Evans and Evans, 1999; Di Bitetti, 2003; Gros-Louis, 2004; Slocombe and Zuberbühler, 2006; Kitzmann and Caine, 2009; Fedurek and Slocombe, 2013; Schel et al., 2013; Kalan et al., 2015), a debate also relevant for questions about animal social awareness and cognitive precursors to language (Fitch, 2005; Tomasello, 2008).

Despite much cross-disciplinary interest, however, comparatively little is known about the evolutionary emergence of food-associated calls in animal communication. Importantly, not all social species produce food-associated calls but, in species where the behaviour has been reported, two main functions have been proposed. A first one states that calls advertise a food resource to other group members, a seemingly altruistic act and a form of food sharing. This is largely because food-associated calls tend to attract others while there appears to be no direct benefit to the caller (Clay et al., 2012; Fedurek and Slocombe, 2013). This hypothesis requires either evidence for enhanced inclusive fitness (Hauser and Marler, 1993a,b; Judd and Sherman, 1996), for instance *via* reduced predation risk or vigilance costs [Clay et al., 2012; birds: Sridhar et al. (2009); red-bellied tamarins: Caine et al. (1995); greater spear-nosed bats: Wilkinson and Boughman (1998)]. Other direct benefits may be in terms of increased foraging success. For example, by attracting conspecifics some species of flocking birds increase the chance of tracking insect swarms (Brown et al., 1991), while ravens and bats attract others to cooperatively defend a food resource against competitors (Heinrich and Marzluff, 1991; Wilkinson and Boughman, 1998). Further benefits concern a lowered risk of conflict by increasing predictability (Fedurek and Slocombe, 2013) and this may even lead to reproductive advantages for a caller (Marler et al., 1986; Evans and Marler, 1994; Van Krunkelsven et al., 1996; Pizzari, 2003; Dahlin et al., 2005). As always, several functions may be acting jointly. In chimpanzees, for example, it has been argued that the production of food-associated calls raises a caller’s social status and also secures cohesion with the rest of the travelling party (Slocombe and Zuberbühler, 2006). Other studies have shown that advertising food is not necessarily altruistic, for example, if the benefits that arise from advertising food are a by-product of others’ selfish behaviours (Connor, 1995). In bottlenose dolphins, for example, there is evidence that role-specialised foraging behaviour is a cooperative by-product mutualism, where participants obtain benefits as a result of the actions of others (Gazda et al., 2005; Gazda, 2016).

The second main theory argues that food-associated calls function in the opposite way, that is, to repel group members from the food source by signalling ownership and an intention to defend it (Caine et al., 1995; Boinski and Campbell, 1996; Gros-Louis, 2004). White-faced capuchins (*Cebus capucinus*), for example, produce “huhs” when approaching a food patch, or when already feeding and a higher-ranking individual approaches them. Subjects are less likely to receive aggression from higher-ranking individuals when calling than when remaining silent (Gros-Louis, 2004). A repelling function has also been proposed for the “coo” calls of rhesus macaques [*Macaca mulatta*; Hauser and Marler (1993b)] and “who” calls in ravens (*Corvus corax*) (Bugnyar et al., 2001). However, it has also been shown that, depending on their age and sex, ravens can use food-associated calls to advertise feeding opportunities (Sierro et al., 2019). In another study, pied babblers remained distanced from speakers playing close-calls and increased their calling rate while feeding in larger groups, indicating that spacing was crucial for foraging efficiency (Radford and Ridley, 2008). Food-associated calls, under these hypotheses, function to signal ownership, motivation to defend and keep other group members at distance, and not to inform them about food, although this naturally occurs as an unintended by-product.

Although both theories predict opposite underlying motivations, they conceptualise food-associated calls as specifically evolved signals to help individuals optimising the cost/benefit ratio when dealing with food. Despite the obvious advantage of possessing such a signal, it is unclear why some species evolved them and others did not. For example, as mentioned earlier, macaques often give ‘coo’ calls in response to food (Mitani, 1986; Hauser and Marler, 1993a), but there are no comparable records for baboons [*Papio* spp.; Silk et al. (2016)], vervet monkeys [*Chlorocebus pygerythrus*; Mercier et al. (2017)] or sooty mangabeys [*Cercocebus atys*; Range and Fischer (2004) and Neumann and Zuberbühler (2016)], despite the fact that they live in somewhat similar societies with presumably similar socio-ecological pressures. For example, sooty mangabeys are terrestrial and live in the same forest habitat as chimpanzees and have a similar social structure as rhesus macaques or white-faced capuchins (multi-male, multi-female groups with some fission-fusion dynamics), species that all produce food-associated calls.

We are not aware of any comprehensive theory that explains why food-associated calls have evolved in some species but not others. An important finding is that, in some species, the acoustic structure of food-associated calls is often similar to the acoustic structures of calls given during close social interactions that do not involve food, usually termed contact or greeting calls. For example, in chimpanzees, the acoustic structure of food-elicited “rough-grunts” (Slocombe and Zuberbühler, 2006) is very similar to the acoustic structure of “pant-grunts,” which are given by lower ranking individuals when a social interaction with a higher ranking one is likely to occur [i.e., “greeting calls,” Laporte and Zuberbühler (2010)]. Another relevant finding in bonobos is that food-elicited “peeps” are also given in non-feeding contexts, such as during grooming, travelling, foraging, or when encountering others, with no obvious acoustic differences (Clay et al., 2015). Therefore, it is likely that acoustically distinct food-associated calls have evolved from more general close-range and individually distinct social calls that draw attention to the caller, while the underlying social functions are similar. Food calls, in other words, may have emerged within the broader category of “close-range” vocalisations by an evolutionary process of acoustic modification and functional specialisation.

In this study, we explore the vocal behaviour during foraging in sooty mangabeys, a forest-dwelling, terrestrial primate that lives in groups of up to 100 individuals, with large group spread. Individuals are often visually isolated from most other group members and spend much time foraging through the forest leaf-litter in search for insects and plant matter, such as the fallen fruits of *Anthonotha* spp., *Saccoglotis gabonensis*, and *Dialium* spp. (Range and Noë, 2002; Janmaat et al., 2006; McGraw et al., 2011). Occasionally, they climb into trees to pick fruits or they consume high-quality but rare foods on the ground, such as eggs, termites, or mushrooms (Range and Noë, 2002; Rödel et al., 2002). Individuals produce two close-range social calls during foraging, ‘twitters’ and ‘grunts’, but also in a range of other situations, including travelling (Neumann and Zuberbühler, 2016) or greeting other group members (Range and Fischer, 2004), but acoustically distinct food-associated calls have not been documented in this species despite years of observation (Range and Fischer, 2004; Neumann and Zuberbühler, 2016).

Nonetheless, it has been observed that, on rare occasions, when sooty mangabeys find highly-valued foods, they can produce calls that appear to instantly attract others to the location [Janmaat et al. (2006), FQ personal observation]. The call is very different from the grunts and strongly resembles vocalisations uttered during fights, a situation that generally attracts bystanders, suggesting that callers anticipate physical aggression when in possession of high-valued foods. Importantly, individuals do not usually call when finding mushrooms, termites, or other high-quality foods, suggesting that the default response is to remain silent.

Our first question concerned the factors that determine grunt production, the most common vocalisation in this species, during the main foraging activity, forest-floor browsing. Our basic theory was that the grunts are the evolutionary raw material from which food calls would evolve. To test this, we predicted that, although mangabeys do not possess food calls, grunt rates should be higher inside food patches than elsewhere, even when not engaged in directed social interactions. We also predicted that, if calling was driven by food competition, call rates should be positively related with the number of potential competitors nearby. Alternatively, if calling was driven by cooperative recruitment rather than competitive spacing, call rates should be negatively related to audience size.

Our second question concerned the factors governing vocal behaviour when finding rare but highly valuable foods, a situation when food calling would be particularly advantageous. Since natural encounters with high quality foods were very infrequent, we carried out a field experiment, during which we let subjects individually encounter eggs in the presence of different audiences. Again, we predicted that if calling was driven by competition, subjects should refrain from calling if no competitors were nearby and the opposite if calling was the product of a cooperative motivation.

## Materials and Methods

### Study Site and Subjects

The study was conducted in Taï National Park in South-western Ivory Coast (5°50′N, 7°21′W), the largest remaining major block of primary forest in West Africa with approximately 454,000 ha of continuous cover. With a mean annual temperature of 24°C, a mean annual rainfall of 1,875 mm (average of 2012–2015; data: Taï Monkey Project) and a distinct dry season in December–January, the park is classified as a ‘tropical moist’ forest (Whitmore, 1990). The study area of about 7 km^2^ was situated near the western border of the park, approximately 20 km southeast of the township Taï. The study group’s home range contained a 2-km^2^ core area where several monkey species had been studied since 1991 as part of a long-term research project (McGraw and Zuberbühler, 2007). The study group has been under constant observation since 1997 and is well-habituated to human observers (Range and Noë, 2002; Quintero et al., 2022). Data collection was mainly during focal animal follows from dawn to dusk over a period of 24 months during different periods: January to May 2013, August 2013 to July 2014 and January to September 2015. See Table 2 for details on experimental trials. During the study period the group size was around 80 individuals.

### Observational Data

Sooty mangabeys produce grunts and twitters during social interactions. The calls are structurally different, but not much is known about their specific functions. Although both calls appear to be given in the same circumstances, we decided to restrict the analyses to grunts only (observational data), mainly because twitters were far less common, the topic of future research. If a grunt was produced during a direct social interaction, that is, when one individual approached another to less than 1 m [see Bernstein (1971) and Range and Noë (2002)], we excluded that event from further analyses, assuming that, in these situations, the calls had a contextually-defined specific social function, such as an invitation for an affiliative interaction (Supplementary Table 1). Hence, our dataset consisted of socially undirected calls only, i.e., when the caller was more than 1 m away from its nearest neighbour. We then scored the subject’s (a) activity (foraging: y/n), (b) location (inside a food patch: y/n) and (c) audience size (number of neighbours within 10 m, the average range of maximum visibility). We predicted that if food calls had a cooperative function, call rates should be high during foraging, inside food patches and with small audiences.

Data collection was in the form of focal animal and instantaneous sampling (Altmann, 1974) on *N* = 33 adult individuals (five males and 28 females). Focal samples were 1 h long and individuals were not sampled twice during the same day. A total of 371 h of focal sampling were carried out on all individuals. During focal animal sampling, data collection included details of each social interaction (Supplementary Table 2) and calling event (Supplementary Table 3) that involved the focal animal. Data from social interactions were used for establishing the dominance hierarchy and social bonds between the individuals. The complete list of behaviours used and a full description for these calculations are described in detail in Quintero et al. (2022). Every 15 min we collected an instantaneous sample, which also included information on the general activity of the focal individual (Supplementary Table 4). We analysed a total of *N* = 1,063 samples collected in time blocks of 15 min. Call discrimination was based on the classification by Range and Fischer (2004). Grunts are short (102–188 ms), low-pitched vocalisations, the most common call type given by sooty mangabeys (Range and Fischer, 2004). When a grunt was produced, we recorded the same variables as the ones recorded every 15 min to be able to compare calling vs. no calling.

### Experimental Data

To test whether sooty mangabeys showed elevated call rates when encountering high quality foods, we experimentally provided high-quality food items (chicken eggs) to a number of subjects in controlled ways (showing the eggs when alone or with neighbours). In each trial, we determined the identities of individuals around the focal animal and documented the subject’s reaction when finding the food. We used chicken eggs due to their similarity with guinea fowl eggs (*Agelastes meleagrides*), which can be found naturally in the forest. The eggs were boiled to avoid transmission of parasites and placed in the projected travel path of a subject, such that it was impossible to form an association between the eggs and the human observers.

### Statistical Analyses

For the observational data, we used generalised linear mixed models with binomial error structure to investigate the variation in call production. We divided each focal sampling session into time-blocks of 15 min. Within each time block we scored whether the focal animal produced at least one grunt (binary, hereafter: call) as the response variable. As mentioned, we only took calls produced in non-directed situations into account. Calls given during grooming, greeting or any other close physical interaction were considered to be part of targeted social interaction. As predictor variables, we included the sex of the focal animal (binary, hereafter: sex), whether or not it was inside a food patch [binary, hereafter: patch type; Range and Noë (2002)], the number of individuals within 10 m (numeric, hereafter: neighbours), the general activity of the focal animal (Binary: foraging or not; hereafter: activity), the presence or absence of socially important individual [“friends”; defined by a DSI score > 1; binary, hereafter: friend; Silk et al. (2013)]. For calculating the DSI we used the following behaviours as variables: “approach,” “inspection,” “presenting groom,” “contact,” “groom,” “handle baby,” and “hug” (Supplementary Table 1). High values of DSI (above 1) indicated for example, regular grooming partners, while low values of DSI (below 1) indicated dyads that rarely groomed, amongst other behaviours. The social status of the focal individual was assessed by its Elo-rating score [numeric, hereafter: Ranking; Neumann et al. (2011)]. Elo-rating scores varied from 1,542 for the highest ranking individual (Norm, the dominant male) to 568 (Tatiana, the lowest ranking female). Finally, the position within the group was scored as central or peripheral [binary, hereafter: position; Range and Noë (2002)]. Observer ID was also included on the models as a fixed factor to control for possible observer differences in data collection (*N* = 2 observers. Binary, hereafter: observer).

In a first model (Supplementary Appendix 5), we addressed the possibility that the number of neighbours affected calling when inside but not outside a food patch (patch * neighbours interaction). We also found it plausible that individuals with high social status were more likely to vocalise than individuals with low social status, but again only inside a food patch (ranking * patch interaction). Third, we addressed the possibility that any status effect on calling might be modulated by the number of neighbours (ranking * neighbours interaction), fourth, we addressed the possibility that the number of neighbours only affected calling when foraging (activity * neighbours interaction), five, we addressed the possibility that the number of neighbours affected calling when in the centre of the group or in the periphery (position * neighbours) and six, we addressed the possibility that the position in the group affected calling when inside, but not outside a food patch (position * patch).

We included random intercepts for focal subject ID and date of observation and added uncorrelated random slopes. Specifically, for focal ID, we included random slopes for all fixed terms in the model that varied within individuals and that represented our main variables of interest (patch, number of neighbours, activity). We did not include random slopes for Elo-rating because we used only Elo-ratings at the end of the study period, i.e., ratings did not change within individuals.

We then built an “informed null model” (Supplementary Appendix 6), which comprised all fixed terms except those that included the three main predictors. The random structure was identical to the full model and we then compared these models with a likelihood ratio test (Dobson, 2002). If the comparison of full and null model revealed a significant difference, we explored the full model with regards to the predictors of interest (i.e., those in the full but not the null model).

Once we assessed the significance of the full model, we tested the interactions. We used R version 3.3.3 (R Core Team, 2017) for the analyses above mentioned, with the glmer function, “lme4” package (Bates et al., 2015) for the GLMMs.

For the experiments, we used generalised linear mixed models with binomial error structure to investigate whether the subjects did or did not call when finding the eggs. For every trial we scored whether the focal animal produced a grunt or a twitter (binary, hereafter: call) as the response variable. As predictor variables, we included the number of individuals within 10 m (numeric, hereafter: neighbours), the time in seconds that it took for another individual to approach the focal animal (numeric, hereafter: time 1st arrival) and whether or not the focal individual was chased after finding the egg (binary, hereafter: chased). We also included random intercepts for focal subject ID. We used conditional inference trees (Hothorn et al., 2006) to select the most significant variables.

### Ethical Note

The experiments replicated natural events (guinea fowl eggs resemble chicken eggs) and we did not interfere with the animals’ normal daily routines, in line with the Animal Behaviour Society Guidelines for the Use of Animals in Research. Research permission and ethical clearance were granted by the Ministère de la Recherche Scientifique et Technique de Côte d’Ivoire.

## Results

### Foraging Behaviour (Observational Data)

**FIGURE 1:**
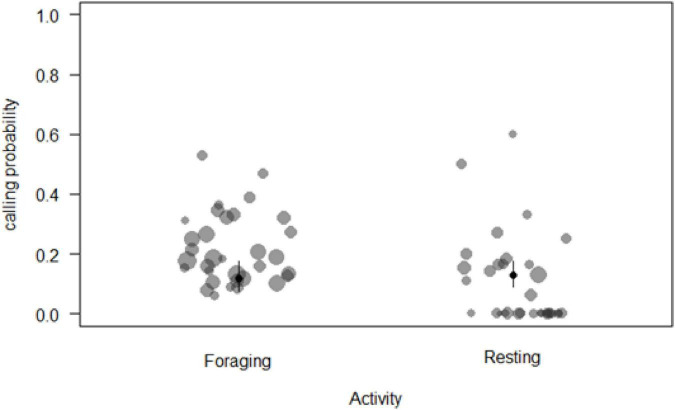
Probability of call production by a focal animal during two main daily activities (circle sizes proportional to the number of samples per individual).

**FIGURE 2:**
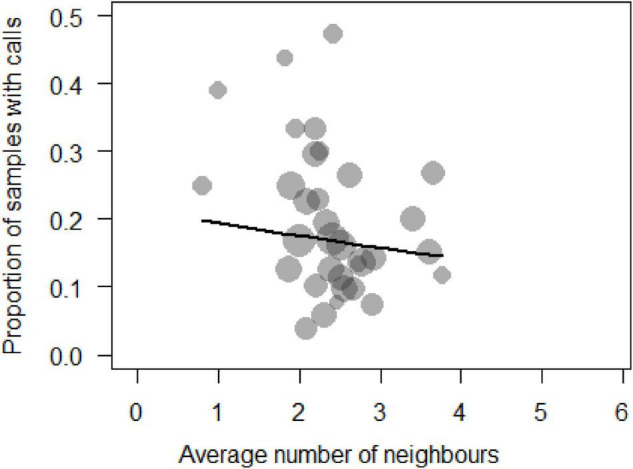
Probability of call production by a focal animal expressed as a function of the number of neighbours within 10 m (Circle sizes are proportional to the number of samples per individual).

We analysed a total of *N* = 1,063 samples (time blocks of 15 min) collected from 33 individuals during 71 days of observation to determine the main factors influencing call production near food patches. The full model was significantly different from the null model (χ^2^ = 33.87, df = 11, *p* = 0.003). We thus proceeded to explore it with regards to whether individuals were inside or outside a food patch, activity and number of neighbours (Supplementary Table 8). We removed five of the six interactions as they were non-significant. These changes resulted in the final model (Table 1), which indicated that sooty mangabeys were more likely to produce grunts in a 15 min sampling period if they were foraging (beta ± SE = 0.61 ± 0.231, *p* = 0.008; Figure 1), if they had fewer neighbours (beta ± SE = −0.17 ± 0.07, *p* = 0.026; Figure 2), if they were in the periphery of the group (beta ± SE = 0.69 ± 0.2, *p* = 0.001) and, finally, if there were with fewer neighbours while being in the periphery of the group (beta ± SE = 0.292 ± 0.116, *p* = 0.012).

**TABLE 1:** Results of the final model for the observational data.

Variables	Estimate	SE	Z	Pr(>| z|)
(Intercept)	3.541	0.667	–5.309	0
Sex	0.375	0.607	0.617	0.536
Observer	0.500	0.330	1.515	0.129
Forage	0.608	0.231	2.630	**0.008**
Friend	0.116	0.267	0.437	0.662
Neighbours	–0.174	0.078	–2.214	**0.026**
Inside food patch	0.394	0.310	1.269	0.204
Ranking	0.092	0.187	0.493	0.622
Position	0.695	0.207	3.348	**0.001**
Neigh * Position	0.292	0.116	2.503	**0.012**

*Interaction between the two variables. The bold values are the significant values.

**TABLE 2:** Results of the final model for the food experiments.

Variables	Estimate	SE	Z	Pr(>|z|)
(Intercept)	4.016	1.933	2.078	0.037
Chased	–4.4174	1.823	–2.422	**0.015**
Neighbours	–1.3793	0.8287	–1.664	0.096

*The bold value is the significant value.*

### Encountering Rare Foods (Experimental Data)

We conducted *N* = 23 experimental trials during which we presented a single chicken egg (Supplementary Table 7) to 11 focal individuals. Subjects called on nine occasions when others approached (less than 5 m) to them and only on three occasions when they were alone. Subjects produced calls in 12 of 23 trials (52.2%). In *N* = 5 further trials, we offered a large number of eggs (>5), thus creating a situation where high-valuable food could not be monopolised. No subject ever vocalised in these situations (0.0%). Importantly, calls produced after finding food did not lead to approaches by others. In all but one occasion, when the subjects were chased after finding food, the finders responded by climbing about 10 m into a tree to escape and eat the eggs alone (Supplementary Video 3). As expected with only one egg, there was no evidence for food sharing or tolerance. Other individuals sometimes stayed close to eat pieces of eggshell dropped by the finder, or to lick leaves covered with egg leftovers.

**FIGURE 3:**
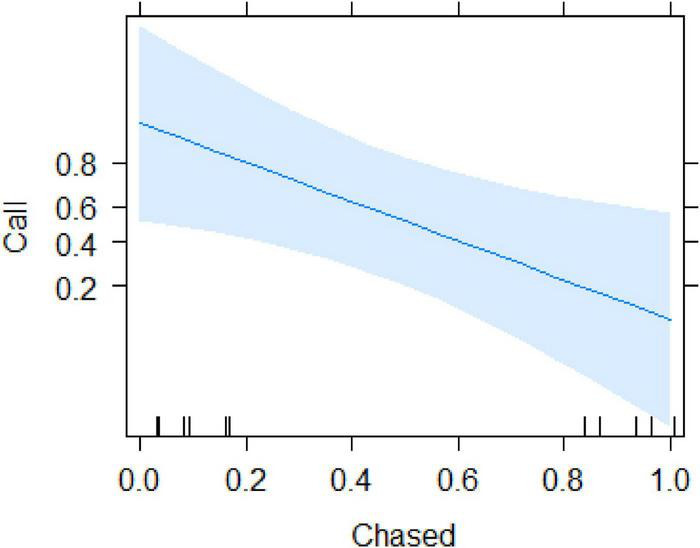
Probability of call production by the subject as a function of whether it was chased or not.

We recorded all social interactions and found that call production was best explained by whether or not the subject experienced physical aggression (i.e., being chased). Using a conditional inference tree we found that calling was only determined by whether or not the subject was chased after finding the food (Supplementary Appendix 9). Specifically, after removing the variable “time 1st arrival,” the resulting model (Table 2) indicated that calling only occurred in cases where the focal animal that found the food was not chased (beta ± SE = −4.41 ± 1.823, *p* = 0.015; Figure 3 and Supplementary Videos 1, 2). Audience size was not significant (beta ± SE = −1.37 ± 0.82, *p* = 0.096).

## Discussion

We were interested in how a social primate that does not possess an acoustically distinct food call uses its vocal behaviour when encountering food, particularly in relation to the two main theories of animal food calls, which make opposite predictions regarding the role of the audience. The cooperative recruitment hypothesis predicts that subjects discovering high-valued foods should call more if alone compared to when surrounded by many of individuals. The competitive hypothesis predicts the opposite, i.e., subjects should call more if in the presence of competitors that are likely to compete over access.

From the observational data, the variables that accounted for most of the variation in calling rates were the number of neighbours, the caller’s own activity and whether the caller was inside a food patch (Table 1). We also found an interaction between the position in the group and the number of neighbours, with subjects more likely to call in the periphery and with small audiences. These findings are in line with the cooperative recruitment hypothesis and do not suggest that calls were uttered as a means to compete over food access. Sooty mangabeys usually forage by browsing through the forest leaf litter of the forest floor, which creates little competition between individuals and only occasionally climbing into trees to harvest fruits or flowers. In the experiments, involving a high-valued food source, we found the same pattern insofar as subjects also vocalised regularly when discovering the food, provided that they were not chased by others (Table 2, Figure 3,and Supplementary Videos 1, 2).

Our data suggest that sooty mangabeys, a species with no food calls, uses its close-range social calls in ways similar to how a cooperatively food-calling species uses its acoustically distinct food calls. If correct, this suggests that “grunts,” the most common call type of sooty mangabeys, represent an ancestral condition in primate vocal evolution, in which food calls and social calls have not diverged acoustically over evolutionary time; something that did happened in chimpanzees, bonobos, and various capuchin monkey vocal evolution. Instead, mangabeys produce the same call type in feeding and non-feeding contexts, although call production patterns are in line with the prediction of the cooperative hypotheses of food calling. In the experiments, we did not find evidence that calls attracted others to the food although, as always with negative evidence, we remain cautious with drawing firm conclusions.

In species with acoustically distinct food calls, the general finding is that these calls are more likely produced to high-valuable foods, such as ripe fruit [chimpanzees: Fedurek and Slocombe (2013); White-face capuchins: Boinski and Campbell (1996)], which are usually consumed in distinct feeding bouts, something that sooty mangabeys rarely do. Another finding in species with food-associated calls is that call production is governed by the presence of others, usually at the onset or during a feeding bout. As stated, sooty mangabeys do not have distinct feeding bouts, but spend most of the day browsing the forest floor to feed on low-quality foods (Janmaat et al., 2006; McGraw et al., 2011). Here, we found increased call rates when foraging (as opposed to moving or resting; Figure 1 and Table 1). We take this finding as supporting the general view that the evolution of food calls may have taken place as an acoustic diversification of more general contact calls, provided it was especially beneficial for a caller to do so when foraging.

As mentioned, amongst the two main functional theories of food calling our data are more in line with the cooperative recruitment theory than the competitive spacing theory. Subjects in the observational dataset were more likely to call with smaller audiences (Figure 2 and Table 1), in line with a function to attract a manageable number of co-feeders in species that do produce acoustically distinct calls. In the experiments, they called more when they were not chased, regardless of the number of neighbours, again suggesting that calls are not used to competitively secure access. Nonetheless, we would not expect individuals to produce food calls (to attract or to repel others) when feeding on ordinary foods, such as *Anthonotha* spp., *Saccoglotis gabonensis* or *Dialium* spp., but when finding valuable foods. In line with this, we found that call rates were higher when foraging, with smaller audiences and when in the periphery, suggesting that grunts in this species function as a context-unspecific contact call. Unfortunately, naturalistic data on encounters with valuable foods, such as mushrooms or termites, were scarce which prevented a systematic analysis. In the experiments, we found that individuals called significantly more when they were not chased regardless of the number of neighbours and, surprisingly these calls never attracted other individuals. This finding is even more striking when taking into account that the eggs were monopolisable in most of the experiments, yet individuals never produced any call when the number of eggs was more than two.

We found no interactions between some of the main variables, particularly between audience size and being inside a food patch, suggesting that calling patterns were similar inside and outside food patches but augmented by the presence of food. A likely reason for this generalised function of sooty mangabey grunts is that they might function to seek contact (Range and Fischer, 2004), a pattern that also emerged during the experiments. Here, subjects called either directly in response to finding the food or delayed when approached by another individual, provided there was no physical aggression, suggesting callers attempted to establish social contact but, since this did not trigger immediate approaches, listeners were probably not able to make inferences about the event. This is different, for example, in chimpanzees where pant grunts (contact calls) are acoustically similar from rough grunts (food calls) (Slocombe and Zuberbühler, 2006; Laporte and Zuberbühler, 2010).

### Evolutionary Transitions to Food Calls

Why do sooty mangabeys produce calls to food in ways that would make them suitable as referential signals, but have not evolved the necessary context-specific acoustic features? Our theory is that a species’ dietary habits and niche specialisation are the main evolutionary driver of acoustically distinct food calls. Mangabeys forage by ingesting large amounts of low-quality foods, mainly collected on the forest floor, while monopolisable high-value foods are rarely consumed, in contrast to chimpanzees and other primates that specialised on high-quality fruits. Most likely, signalling the discovery of low-quality foods is of no adaptive significance, while individuals are almost always surrounded by other group members, hereby removing two main sources of call evolution. Announcing ownership is equally futile, as the food is abundant and found in large patches that cannot be monopolised (Range and Noë, 2002; McGraw et al., 2011), again in contrast to fruit trees where feeding space and availability is limited. Some social structures, such as matrilineally-based hierarchies with stable dominance relations, may further remove the potential for conflicts, as it is the case in mangabeys but not chimpanzees.

In conclusion, although sooty mangabeys call when finding high-value foods, they do so not because they want to inform others or to claim ownership, but because of a general motivation for social cohesion, allowing listeners to know the caller’s whereabouts (Schamberg et al., 2018) and reduce the risk of group fission, which is always high when individuals stop to feed, a situation also created by our experiments. Nevertheless, calling patterns were identical to species that cooperatively denote food sources, suggesting that sooty mangabeys possess calling patterns that are suitable for the evolution of food-specific vocalisations.

## Data Availability Statement

The raw data supporting the conclusions of this article will be made available by the authors, without undue reservation.

## Ethics Statement

The animal study was reviewed and approved by Ministère de la Recherche Scientifique et Technique de Côte d’Ivoire.

## Author Contributions

FQ and KZ: study design and interpreted and drafted the manuscript. ST, MM, and FQ: data collection. FQ: statistical analysis. KZ: provision of necessary tools and resources. All authors read and approved the final manuscript.

## Conflict of Interest

The authors declare that the research was conducted in the absence of any commercial or financial relationships that could be construed as a potential conflict of interest.

## Publisher’s Note

All claims expressed in this article are solely those of the authors and do not necessarily represent those of their affiliated organizations, or those of the publisher, the editors and the reviewers. Any product that may be evaluated in this article, or claim that may be made by its manufacturer, is not guaranteed or endorsed by the publisher.
