# Supplementary material for: The Evolution of Food Calls: Vocal Behaviour of Sooty Mangabeys in the Presence of Food
Source: Front Psychol. 2022 Jun 30;13:897318. doi: 10.3389/fpsyg.2022.897318 (PMC9282157; doi:10.3389/fpsyg.2022.897318)

**Appendix**

**Supplementary Table 1.**  Ethogram of sooty mangabeys of Taï National Park, Ivory Coast. Adapted from Range & Noë (2002).

|  | **Abbreviation** | **Behaviour** | **Definition** |
| --- | --- | --- | --- |
| Affiliative Behaviours | Co | Contact | two individuals are in contact sitting usually and don't really interact with each other |
|  | Hu | Hug | two individuals are hugging each other (usually around the shoulders or neck) |
|  | Hb | handle baby | individual is manipulating the baby |
|  | Ca | carry baby without stress | individual is carrying a baby to move around (usually on the stomach but sometimes on the back) |
|  | Mtm | mouth to mouth | individual is smelling a partner's mouth and vice versa |
|  | Lm | lips smacking | individual is making noise by clapping its lips (usually while grooming or approaching a subordinate) |
|  | Pl | play (general) | individual is playing (no description) |
|  | Gr | Groom | individual is grooming a partner |
|  | Pr | prensenting groom | individual is presenting a part of its body to be groomed (usually elbow, head or back) |
|  | nas | non-agonistic supplant | individual is approaching another animal who is occupying a resource and replaces that individual without overt aggression |
|  | Ap | approach | individual is approaching another animal (r<2m) |
|  | To | Touch | individual touch a partner in a gentle way, hand on shoulder |
|  | Ins | Inspection | Individual inspects another individual sexual parts. |
|  | Fo | Follow | individual is following another animal |
|  | Le | Leave | individual is leaving the proximity of another (perimeter of three meters) |
|  | Ipl | invitation to play | initiation of play (show teeth, look upside down, quick touch before darting and jumping) |
| Agonistic Behaviours | Fi | Fight | two partners stand up and slap each other |
|  | asf | attempt stealing food | individual is trying to steal another's food |
|  | akb | attempt kidnapping baby | individual is trying to steal a mother's baby |
|  | Bt | bared teeth | individual is showing its teeth (usually while approaching a dominant) |
|  | Av | avoidance | individual is avoiding another individual who is approching it |
|  | Jp | jump aside | individual jump aside to avoid a partner, an aggression, something scary |
|  | Cr | crawl on the ground | individual is lying on the ground while looking at its aggressor |
|  | Fl | Flee | individual is fleeing an aggressor |
|  | Lo | look around for support | individual is searching around him for support while it is threatened by another |
|  | cas | carry baby with stress | individual is carrying a baby to escape a stressful situation for itself or for the baby (infanticide attacks) |
|  | St | Stare | individual is threatening (look / go forward) |
|  | Stl | stare and lunge | individual is threatening and half attacking (go forward / backward) |
|  | Gp | Grip | individual is gripping the other's fur |
|  | Bi | Bite | individual is bitting |
|  | Ch | Chase | individual is chasing another |
|  | Sf | stealing food | individual is stealing another's food |
|  | Tp | take place | individual takes another's place |
|  | Kb | kidnapping baby | individual managed to steal a baby and refuse to hand it back to its mother |
|  | Ign | ignore / turn back | individual ignore a partner's sollicitation (for grooming, mating, nursing, etc) and even can turn its back |
|  | Sa | support aggressor | individual supports the aggressor in a conflict |
|  | Sv | support victim | individual supports the victim in a conflict |

**Supplementary Table 2**. Variables collected during focal animal sampling

| **Datum** | **Description** |
| --- | --- |
| **Date** | Date when the encounter took place |
| **Time** | Time when the encounter took place |
| **Distobserver** | Approximate distance from the observer to the focal animal |
| **Height** | Height of the animal at the moment of the encounter or interaction |
| **BehaviorCategory** | Whether the behavior was Affiliative, Aggressive, Sexual or Neutral |
| **IDFocal** | Name of the focal individual |
| **IDEncountree** | Name of the individual encountered |
| **1st Behaviour** | First behaviour that indicates the direction of the encounter (approach/being approached) |
| **2nd Behaviour** | Behaviour after the focal individual approaches or is approached by the encountered individual |
| **In/outofsight** | Whether the encountered individual was insight or out of sight for the focal individual at least 20 seconds before the encounter |
| **VocalizationFocal** | Whether the focal individual vocalizes during the encounter |
| **TypeVocalizationFocal** | Type of vocalization emitted by the focal individual |
| **VocalizationEncountree** | Whether the encountered individual vocalizes during the encounter |
| **TypeVocalizationEncountree** | Type of vocalization emitted by the encountered individual |
| **DistbtwnID** | Distance between the focal individual and the encountered individual |
| **NIDin1m** | Number of individual within 1m around the focal individual at the moment of the encounter |
| **NIDIn5m** | Number of individual within 5m around the focal individual at the moment of the encounter |
| **NIDIn10m** | Number of individual within 10m around the focal individual at the moment of the encounter |
| **IDin1m** | Names of the individuals within 1m around the focal individual at the moment of the encounter |
| **IDin5m** | Names of individuals within 5m around the focal individual at the moment of the encounter |
| **IDin10m** | Names of individuals within 10m around the focal individual at the moment of the encounter |
| **in/out food patch** | Whether the encounter takes place inside or outside a food patch |
| **Kind of patch** | The kind of food patch |
| **Remarks** | Any interesting event that was not included on the taken data |

**Supplementary Table 3**. Data collected in conjunction with vocal behaviour

| **Datum** | **Description** |
| --- | --- |
| **Date** | Date of the vocalization |
| **Time** | Time of the vocalization |
| **Observer** | Name of the observer |
| **FocalID** | Name of the focal individual |
| **Activity** | General activity of the focal individual (Resting, moving, foraging, social) |
| **Vocalization** | Type of vocalization emitted by the focal individual |
| **Nbin1m** | Number of individual within 1m around the focal individual |
| **Nbin5m** | Number of individual within 5m around the focal individual |
| **Nbin10m** | Number of individual within 10m around the focal individual |
| **ID1mbefore** | Names of the individuals within 1m around the focal individual |
| **IA5mafter** | Names of individuals within 5m around the focal individual |
| **IA10mafter** | Names of individuals within 10m around the focal individual |
| **Remarks** | Any interesting event that was not included on the taken data |

**Supplementary Table 4**. Data collected during instantaneous sampling

| **Datum** | **Description** |
| --- | --- |
| **Date** | Date of the instantaneous sampling |
| **Time** | Time of the instantaneous sampling |
| **Observers** | Name of observer collecting the data |
| **Weather** | General description of the weather at the forest (Rainy, cloudy, sunny) |
| **Disttoobserver** | Approximate distance from the observer to the focal animal |
| **FocalID** | Name of the focal individual |
| **Activity** | General activity of the focal individual (Resting, moving, foraging, social) |
| **Height** | Height of the animal at the moment of the encounter or interaction |
| **PositionInGroup** | Relative position of the focal individual to the rest of the group. Definition by Range & Noë (2002) |
| **NearestNeighbour** | Name of closest individual to the focal |
| **DistanceNearest** | Distance in meters from the focal to the closest neighbor |
| **Nb1m** | Number of individual within 1m around the focal individual |
| **Nb5m** | Number of individual within 5m around the focal individual |
| **Nb10m** | Number of individual within 10m around the focal individual |
| **IndIn1m** | Names of the individuals within 1m around the focal individual |
| **IndIn5m** | Names of individuals within 5m around the focal individual |
| **IndIn10m** | Names of individuals within 10m around the focal individual |
| **In/Out of patch** | Whether the encounter takes place inside or outside a food patch |
| **Kind of patch** | The kind of food patch |
| **Remarks** | Any interesting event that was not included on the taken data |

**Supplementary appendix 5.** Full model for the observational data

Call ~ sex +observer + forage + friend + patch*neighbours + ranking*patch + ranking*neighbours + activity*neighbours + position*neighbours + position*patch

+(1|focal ID) +(1|date)

**Supplementary appendix 6.** Null model for the observational data

Call ~ sex + observer + ranking

+(1|focal ID) +(1|date)

**Supplementary Table 7.** Results of the food experiments

| Trial | Date | ID | Call | Call type | Chased | Approached |
| --- | --- | --- | --- | --- | --- | --- |
| 1 | 28.07.2013 | Nina | 0 | NA | 1 | 0 |
| 2 | 27.10.2013 | Libia | 1 | grunt-twitter | 0 | 0 |
| 3 | 09.07.2015 | Libia | 0 | NA | 1 | 0 |
| 4 | 26.05.2014 | Oval | 0 | NA | 1 | 0 |
| 5 | 05.06.2014 | Hilda | 0 | NA | 1 | 0 |
| 6 | 18.09.2015 | Fiji | 1 | grunt-twitter | 1 | 0 |
| 7 | 20.08.2015 | Fiji | 0 | NA | 0 | 0 |
| 8 | 06.08.2015 | Fiji | 1 | Grunt | 0 | 1 |
| 9 | 12.06.2014 | Tatiana | 1 | Grunt | 0 | 0 |
| 10 | 22.06.2014 | Mona | 0 | NA | 1 | 0 |
| 11 | 23.07.2015 | Fiona | 1 | Grunt | 0 | 1 |
| 12 | 29.07.2015 | Teresa | 1 | Grunt | 0 | 1 |
| 13 | 26.08.2015 | Agatha | 1 | grunt-twitter | 0 | 0 |
| 14 | 13.09.2015 | Johanna | 1 | Grunt | 0 | 1 |
| 15 | 18.11.2015 | Fiji | 1 | Grunt | 0 | 1 |
| 16 | 19.11.2015 | Tatiana | 0 | NA | 1 | 0 |
| 17 | 20.11.2015 | Tatiana | 1 | grunt-twitter | 0 | 1 |
| 18 | 24.11.2015 | Fiji | 0 | NA | 0 | 0 |
| 19 | 17.12.2015 | Nina | 1 | grunt-twitter | 0 | 1 |
| 20 | 18.12.2015 | Mona | 0 | NA | 1 | 0 |
| 21 | 19.12.2015 | Mona | 1 | Grunt | 0 | 1 |
| 22 | 27.01.2016 | Agatha | 0 | NA | 0 | 0 |
| 23 | 06.02.2016 | Fiji | 0 | NA | 0 | 0 |

**Supplementary Table 8.** Results of the observational data

| Variables | Estimate | SE | | | Z | Pr(>\|z\|) | | |
| --- | --- | --- | --- | --- | --- | --- | --- | --- |
| (Intercept) | -4.131 | 0.794 | | | -5.201 | 0.000 | | |
| Sex  Observer  Forage  Friends  Neighbours  Inside Food Patch  Ranking  Position  Neighbours:Patch  Patch:Ranking  Neighbours:Ranking  Neighbours:Position  Patch:Position  Forage:Neighbours | 0.432  0.481  0.564  0.118  -0.332  1.023  0.171  1.301  0.213  -0.095  -0.025  0.323  -0.694  -0.063 | | 0.622  0.335  0.244  0.269  0.206  0.527  0.299  0.573  0.215  0.293  0.060  0.120  0.602  0.145 | 0.695  1.434  2.304  0.441  -1.610  1.939  0.574  2.272  0.992  -0.325  -0.424  2.679  -1.153  -0.436 | | | 0.487  0.151  0.021  0.658  0.107  0.052  0.566  0.023  0.321  0.745  0.671  0.007  0.248  0.663 |  |

**Supplementary appendix 9.** Conditional inference tree results for the rare food encountering model


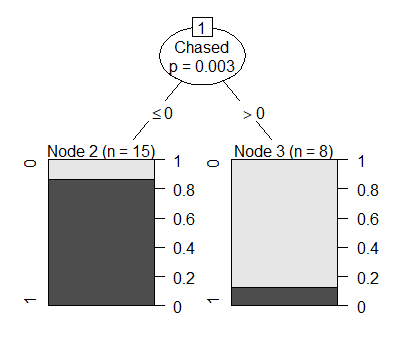

Supplement: Supplementary file 1 [file Data_Sheet_1.docx]
